# A full review of online OPAT educational resources for patients and healthcare professionals in the grey literature revealing resource gaps

**DOI:** 10.1093/jacamr/dlag192

**Published:** 2026-09-01

**Authors:** C Watson, J Spencer-Jones, K Heard, A Jenkins, T P Gauthier, B A Rogers, A N Marizan Nor, F B Sime, M Gilchrist

**Affiliations:** Pharmacy Department, London North West University Healthcare NHS Trust, London, UK; Pharmacy Department, Mid Yorkshire Hospitals NHS Trust, Wakefield, UK; Pharmacy Department, University Hospitals Plymouth NHS Trust, Plymouth, UK; Pharmacy Department, University Hospitals Birmingham NHS Foundation Trust, Birmingham, UK; Clinical Pharmacy Enterprise, Baptist Health South Florida, Miami, FL, USA; Monash Infectious Diseses, Monash Health, Melbourne, Victoria, Australia; Medical Department, Hospital Sungai Buloh, Selangor, Malaysia; School of Pharmacy and Pharmaceutical Sciences, The University of Queensland, Brisbane, Australia; Department of Pharmacy/Infection, Imperial College Healthcare NHS Trust, London, UK; Department of Infectious Diseases, Imperial College London, London, UK

## Abstract

**Background:**

There is an absence of practical educational resources for both healthcare professionals and patients, which are crucial to support safe and consistent delivery of Outpatient Parenteral Antimicrobial Therapy (OPAT) across health systems.

**Objectives:**

To identify and appraise high-quality, free-to-access educational resources available in the English language across grey literature to support OPAT service delivery for healthcare professionals and/or patients.

**Methods:**

This review searched three grey-literature databases, conducted a targeted web search, reviewed six organizational websites and consulted international experts in OPAT service delivery to identify free-to-access educational resources published in the English language to address the educational needs of healthcare professionals and patients. Study investigators identified a final list of resources based on applied inclusion and exclusion criteria and categorized as peer-reviewed accredited, non-accredited or expert-opinion-based materials.

**Results:**

A total of 36 potential resources were identified, of which nine were selected for review. Resources were grouped into four categories; webinars and conference archives, expert-led service overviews, patient-facing information and online courses for healthcare professionals. Only two patient-directed OPAT educational materials were identified and these were based on expert opinion rather than formally accredited materials. Most resources were developed within high-income healthcare systems but offer adaptable principles that can support OPAT practice development in low- and middle-income country settings.

**Conclusions:**

The lack of readily available resources aimed at general OPAT information represents a missed opportunity in patient care and education. Addressing these educational gaps is essential to support safe, effective and sustainable OPAT service delivery and improve patient outcomes.

## Introduction

Since its inception in the 1970s, Outpatient Parenteral Antimicrobial Therapy (OPAT) has become an internationally recognized modality of care.^1^ OPAT services allow medically stable patients to be discharged from the hospital releasing precious inpatient resources under an agreed clinical infection management plan.^1^ The benefits of such a service are not only felt by the patient and their immediate family but also the wider healthcare organization in terms of efficiency in care and flow, along with use of resources and reduced costs.^2,3,4^ Underpinning OPAT services is the multi-disciplinary team, which should include medical, nursing and pharmacy services.^5^ Central to their working is effective education around what OPAT is and what it can provide and support.

Internationally, OPAT services are well-established in many high- and middle-income countries (HMICs), however, there is a growing interest in lower-middle-income countries (LMICs), where OPAT is viewed as a strategy to improve healthcare access and efficiency.^6,7^ Recent healthcare strategies, such as the NHS Fit for the Future: 10 Year Health Plan for England, emphasize shifting care delivery into the community and exploring alternative models for providing parenteral antimicrobials outside hospital settings, even when these services are not formally designated as OPAT.^8^ This reflects a broader global trend towards outpatient parenteral therapy.

While the published literature provides guidance to support OPAT programme development, most evidence is concentrated in HMIC settings and there are still barriers to implementation, including the need for clearer organizational structure and guidelines.^9^ In addition, there is an absence of practical educational strategies for both healthcare professionals and patients, resources crucial to support safe and consistent delivery of OPAT across health systems.

Considering these gaps, this review aimed to identify and characterize free-to-access grey-literature materials to address the educational needs of healthcare professionals (HCPs) and patients. The term ‘grey literature’ is often used to refer to reports published outside traditional commercial publishing channels,^10^ which includes peer-reviewed resources.

## Methods

This scoping review adhered to the Arksey and O’Malley framework, which comprises six stages aimed at systematically addressing the research question.^11^ The Arksey and O’Malley framework was selected as it provides a structured and widely used approach for conducting scoping reviews, particularly when the objective is to map the extent, range and characteristics of available evidence rather than evaluate effectiveness.

### Stage 1: identifying the research question

The purpose of this review is to appraise the current free-to-access educational resources available for OPAT service delivery within the grey literature and answer the research question:

What useful educational resources in English language are available in grey literature for OPAT services directed towards HCPs and/or patients?

The objectives of the review were:

- Identify free-to-access grey-literature resources that assist with educating HCPs or patients on the purpose, set up, implementation or care on an OPAT service.
- Identify strengths, limitations and opportunities for improvement across patient- and professional-facing materials.

### Stage 2: identifying resources

Three approaches were used to identify resources on OPAT:

i. Grey-literature databases

A preliminary group was established, with each researcher (M.G., C.W., K.H., J.S.J., A.J.) acting as search strategists systematically exploring grey-literature databases to identify potential databases and search terms. The group then met to define databases and search terms.

- The following grey-literature databases were agreed: Mednar, OAIster, Open Access Theses and Dissertations.
- The following terms were used to search grey-literature databases as both keywords in the title and/or subject headings, as appropriate: (‘antibiotic’ OR ‘antimicrobial’ OR ‘anti-infective’) AND (‘parenteral’ OR ‘intravenous infusion’ OR ‘home infusion’ OR ‘elastomeric’ OR ‘pump’ OR ‘intramuscular’ OR ‘subcutaneous’) AND (‘outpatient’ OR ‘home’ OR ‘ambulatory’ OR ‘community’ OR ‘discharge’ OR ‘self’ OR ‘care giver’). Additional search terms used to narrow searches if required: (‘education OR ‘learn*’ OR ‘e-learning’).

An educational resource was defined as material specifically designed to facilitate learning, instruction, or training in a particular subject, topic or skill and could be in the format of a textbooks, workbook, handout, worksheet, presentation, video, audio recording, interactive software or an online course.

ii. Web search

For the search strategy, each researcher (M.G., C.W., K.H., J.S.J., A.J.) conducted a web search, reviewing the first 100 hits using any OPAT-education related terms. Less restrictive search terms were used compared to grey-literature databases. Targeted websites included WHO, IDSA, BSAC, SAPG, YouTube and X.

iii. Study investigator nominations

International colleagues were invited to nominate educational resources.

### Stage 3: selection of resources for review

A three-step method was applied for resource selection. In the first step, all researchers independently screened titles and content of citations returned from the grey-literature search. In the second step three researchers (C.W., K.H., M.G.) identified the final list of resources based on applied inclusion and exclusion criteria.

Resources were included if they:

- provided education content aimed at patients or healthcare professionals on OPAT (e.g. explaining OPAT processes, clinical considerations, self-management, antimicrobial therapy principals)
- were accessible online and free-to-access
- were published in English
- were generalizable in content (i.e. not limited to a single local service, organization, or specific product/device).

Resources were excluded if they:

- originated from academic journals, books or other conventional peer-reviewed publication sources, as the review focused on online educational resources specifically designed to support learning and professional development in OPAT
- provided information only (e.g. definitions, service descriptions, marketing material) without an educational component
- focused solely on care of intravenous access device, general medicines administration without specific relevance to OPAT or antimicrobials.

In the final step, the list of resources were categorized according to the following criteria:

- Peer-reviewed, accredited
- Peer-reviewed, non-accredited
- Expert opinion

#### Review process

If any contributors had a conflict of interest such as authorship of a resource, they abstained from review. All remaining resources were then assessed and selected for final review and data extraction.

### Stage 4: data extraction

A standardized data-extraction form was used to capture the characteristics of the included resources. The following information was evaluated in line with an educational review: resource type, source of resource, focus of topic, target audience, relevance and setting of resource.

### Stage 5: data summary and synthesis of results

Data from included resources were descriptively charted following scoping-review methodology. As this review focused on grey literature, findings were interpreted to describe the range, format and target audiences of available OPAT educational materials, and to identify key gaps rather than to appraise effectiveness.

### Stage 6: consultation

Stakeholders from the international OPAT community were consulted to refine the search approach and interpret the findings. Their feedback supported consensus and ensured the results reflected real-world educational needs.

## Results

To refine searches, the three grey literature databases were selected (Mednar, OAIster, Open Access These and Dissertations) but ultimately resulted in no identified resources related to the research question. Three responses from international colleagues (T.G., F.S., B.R.) as study investigator nominations (USA, Australia and Malaysia) were received identifying two resources. Thirty-four resources were identified via web search and targeted websites.

**Figure 1.**
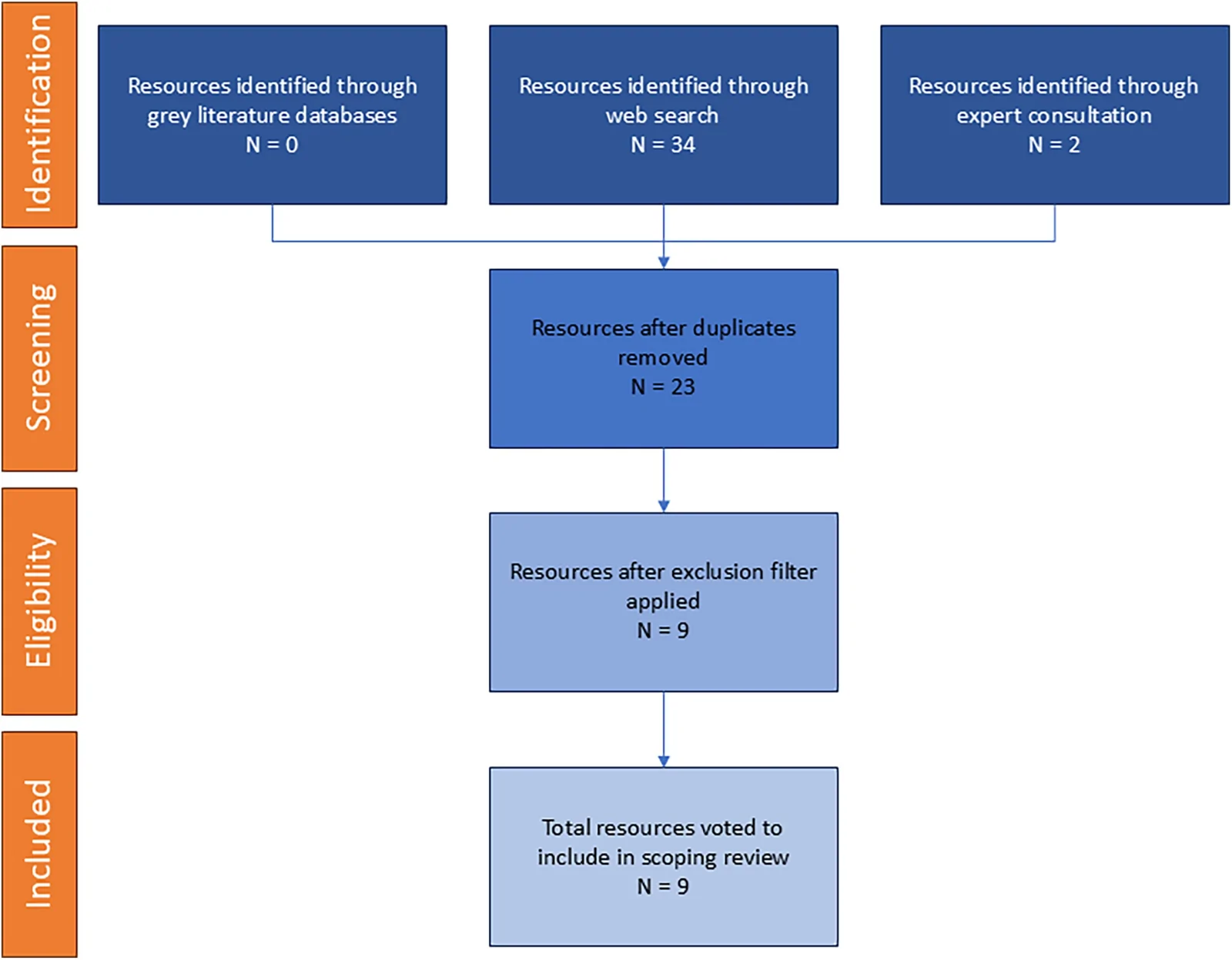
Flow diagram showing the identification, screening and selection of online OPAT educational resources.

The search strategy identified 36 potential resources where nine were selected for review. Resource identification and selection processes are detailed in Figure 1.

**Figure 2.**
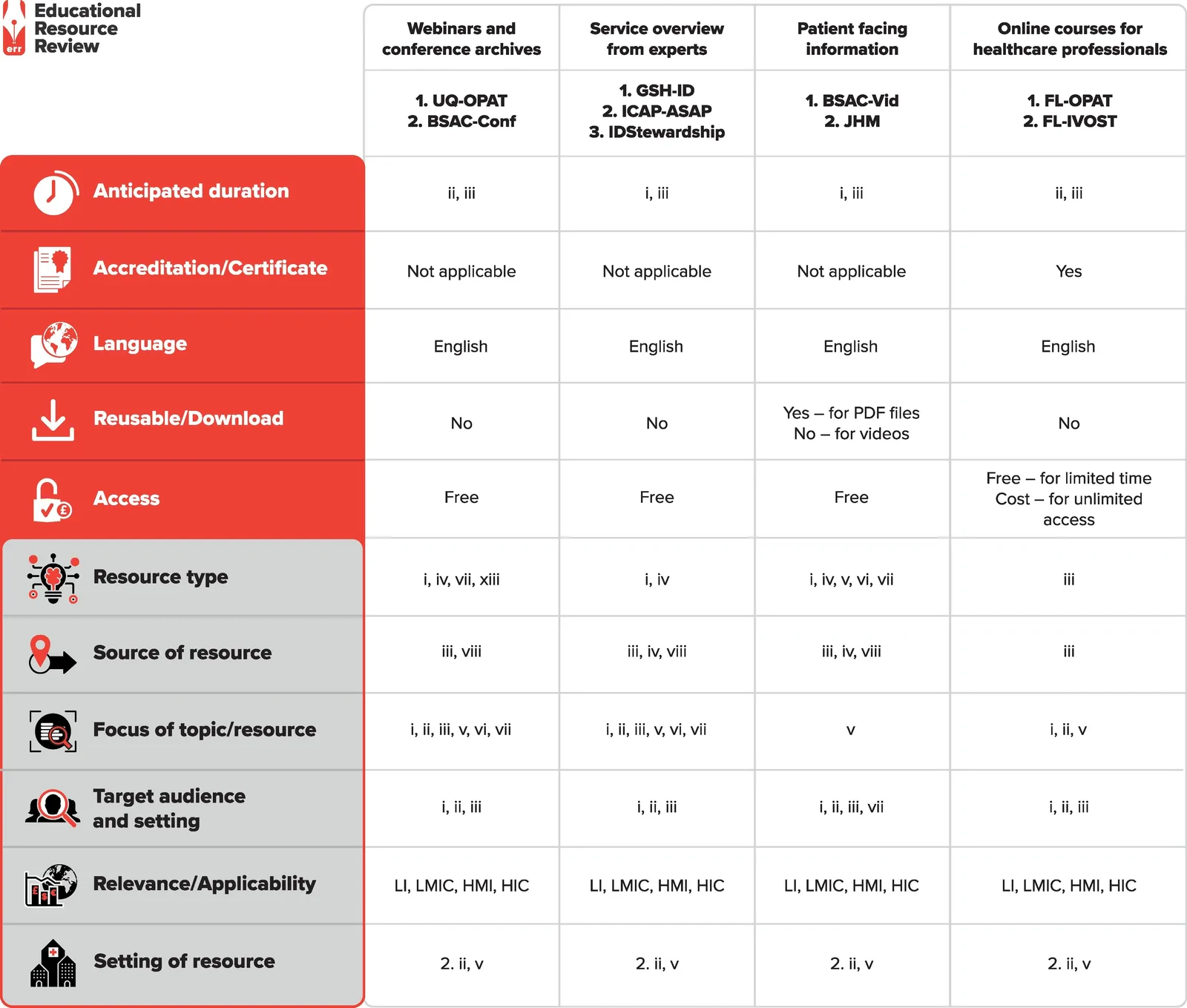
General characteristics of resources included in the scoping review. Anticipated duration: (i) short <4 h; (ii) long >4 h; (iii) self-paced. Resource type: (i) website online reading material and other resource; (ii) website primarily aimed at news items; (iii) online/distance learning courses (massive open online course, unfacilitated courses, online modules)/community of practice; (iv) webinars, video, online lectures, podcasts, animation video, maps, photos; (v) clinical practice antimicrobial stewardship materials: PDFs, PowerPoints, newsletters, infographics, pamphlets, e-portfolios, workbooks; (vi) guidelines, policies, handbooks; (vii) material from face-to-face non-e-learning courses (programmes, teaching materials etc. from workshops, lectures, seminars); (viii) e-books via apps; (ix) public, media, political awareness and engagement materials; (x) commercial advertising (TV, radio, film, social media); (xi) evidence: by systematic reviews/meta-analysis in relation to antimicrobial resistance; (xii) datasets, compelling/illustrative case studies on antimicrobial stewardship; (xiii) patient stories. Source of resource: (i) governments; (ii) professional societies; (iii) universities/higher education institutes; (iv) healthcare facilities; (v) WHO; (vi) industry; (vii) insurance companies; (viii) non-governmental organizations (NGOs). Focus of resource: (i) principles/practice of prudent prescribing; (ii) antimicrobial stewardship principles/practices; (iii) guidelines/policies/pathways for syndrome management of infections; (iv) infection prevention/control; (v) implementation/behaviour change; (vi) evaluation/measurement; (vii) evidence gathering. Target audience: (i) doctors; (ii) pharmacists; (iii) nurses/midwives; (iv) non-medical managers; (v) public health; (vii) laboratory; (viii) infection prevention practitioners. Relevance/applicability: LIC, low-income country; LMIC, low- and middle-income country; HMIC, high- and middle-income countries; HIC, high-income country. Setting of resource: (1) pre-service (university, higher education institution); (2) service: (i) hospital; (ii) outpatient clinic; (iii) community/general practice; (iv) long-term care facility/nursing home; (v) hospital and ambulatory; (vii) other.

Resources were grouped into four categories; webinars and conference archives, service overview from experts, patient-facing information and online courses for healthcare professionals. General characteristics of each resource are described in Figure 2.

### Webinars and conference archives

Two major online sources provide freely accessible OPAT-focused educational recordings delivered by clinical and academic experts.

#### The University of Queensland: Outpatient Parenteral Antimicrobial Therapy, Hospital in the Home masterclasses

The University of Queensland (Australia) hosts an extensive series of OPAT and Hospital-in-the-Home (HITH) Masterclass webinars first produced in 2021.^12^ These recordings are produced through the University’s interdisciplinary infectious diseases and antimicrobial stewardship programmes and are designed for healthcare professionals involved in delivering outpatient IV antimicrobial therapy. The webinar recordings offer expert-led sessions on clinical practice, service models and antimicrobial stewardship. Content is delivered through recorded presentations supported by slides, case discussions and practical guidance. The materials are organized chronologically on the hosting platform.

#### British Society for Antimicrobial Chemotherapy: National OPAT Conference Archives

Similarly, the British Society for Antimicrobial Chemotherapy (BSAC) provides access to a comprehensive archive of its National OPAT Conference recordings dating back to 2013 to 2025.^13^ BSAC is a UK-based professional society involved in antimicrobial stewardship and OPAT good-practice guidance, and its annual conference brings together clinicians, pharmacists, nurses and service leads. The archived presentations cover emerging OPAT evidence, novel service delivery models, programme governance, long-acting antimicrobial agents, paediatric OPAT considerations and multi-disciplinary perspectives on operational challenges. The archive includes keynote lectures, panel discussions and real-world service evaluations. Materials are presented as video recordings accompanied by session information and speaker details.

Both platforms offer static collections of recordings without subject-specific search functionality.

### Service overview from experts

Several resources were identified that provide expert perspectives on OPAT service organization and clinical practice, offering structured overviews of key operational and stewardship principles.

#### GSH infectious diseases: evolution of OPAT in the UK—what lessons can be transferred to South Africa

The Division of Infectious Diseases and HIV Medicine at Groote Schuur Hospital (GSH), University of Cape Town, hosts weekly educational webinars aimed at advancing infectious diseases practice across South Africa and beyond. These webinars cover a broad range of clinical and stewardship-focused topics and are delivered by regional and international experts. One session, produced in 2021, focuses specifically on the evolution of OPAT in the UK, outlining service development, governance structures and clinical pathways, while discussing how these lessons may be adapted for low- and middle-income settings such as South Africa.^14^ Recordings are available as video presentations within an openly accessible archive, accompanied by speaker details and session titles.

#### Nebraska ICAP and ASAP Programs: ‘Don't Standpat on OPAT’—stewardship opportunities at transitions of care

The Nebraska Infection Control Assessment and Promotion (ICAP) programme and the Nebraska Antimicrobial Stewardship Assessment and Promotion Program (ASAP), statewide initiatives supporting healthcare quality, safety and stewardship, host a range of educational webinars supporting infection prevention and antimicrobial stewardship across healthcare settings. Their recorded session related to OPAT focuses in 2022 on stewardship opportunities during transitions of care in OPAT services.^15^ The session is presented as a recorded lecture with supporting slides.

#### ID stewardship: Mediwala KN—an overview of Outpatient Parenteral Antimicrobial Therapy practices

IDStewardship.com, an online platform developed by infectious diseases pharmacists in the USA, to support antimicrobial stewardship education, provides a comprehensive written overview of OPAT practice, covering service structure, operational considerations and antimicrobial stewardship principles.^16^ The webpage published in 2017 summarizes operational elements such as team composition, safety processes and coordination needs during outpatient parenteral therapy. Content is presented as a structured, text-based educational article, supplemented by frequently asked questions and links to related stewardship topics. The material is freely accessible and written for a wide professional audience, including pharmacists, clinicians and trainees.

### Patient-facing information

Two patient-directed OPAT educational materials were identified across international healthcare organizations although these were largely derived from expert opinion rather than formally accredited materials.

#### British Society for Antimicrobial Chemotherapy: OPAT teaching videos for self-administration of IV antibiotics

The BSAC provides a series of OPAT self-administration teaching videos, offering practical demonstrations for patients learning to administer IV antibiotics at home.^17^ The collection, produced in 2022, includes eight medication-specific demonstration videos, covering ceftriaxone, daptomycin, piperacillin/tazobactam, teicoplanin and three meropenem dose formats, as well as ertapenem.^1^ These videos provide step-by-step visual instruction tailored to the antibiotic being administered. The webpage also links to additional service-level videos from partner institutions.

#### Johns Hopkins Medicine: Outpatient Parenteral Antimicrobial Therapy patient education materials

Johns Hopkins Medicine, an academic health system and medical school based in Baltimore, Maryland and part of Johns Hopkins University, hosts a suite of OPAT patient education materials, including written guides and visual resources to support safe outpatient IV antimicrobial administration.^18^ The resource includes seven written guides for patients and healthcare workers, such as the OPAT Introduction, an OPAT Teaching Plan, discharge handouts and multiple checklists covering nursing tasks and supply preparation. The website offers educational materials for bathing with an intravenous line. There are also six instructional videos covering different infusion delivery devices. No publication date was available for these resources.

### Online courses for healthcare professionals

Two accredited online courses designed for healthcare professionals were identified, both offering expert-led, structured education to support competency in OPAT service delivery and prescribing practice.

FutureLearn is an online learning platform that offers a wide range of courses from universities and institutions around the world. It was founded in 2012 and is owned by The Open University in the UK. The platform collaborates with various educational providers to deliver high-quality, accessible learning experiences, often focusing on topics such as business, health, technology and arts. Content is peer-reviewed reviewed by subject-matter experts and continuing professional-development accredited. Courses are typically structured in a way that encourages engagement and interaction among learners. There are two courses produced by the BSAC with a focus on OPAT.

#### FutureLearn: Outpatient Parenteral Antimicrobial Therapy

The first resource, published in 2021, introduces the concept of OPAT, the roles and responsibilities within the multi-disciplinary OPAT team, the importance of patient selection and antimicrobial choice, device selection and monitoring. The course is designed to be completed over 6 hours per week for 2 weeks.^19^

#### FutureLearn: intravenous to oral switch—within outpatient parenteral antibiotic therapy

The second resource, also published in 2021 introduces how oral and complex oral antibiotics can be used in an OPAT setting and the evolving entity of Complex (Outpatient) Oral and Parenteral Antibiotic Therapy (COPAT) services. It details the key practice points for OPAT-based prescribers (e.g. route, choice, duration, recording of information), when intravenous oral antibiotic switch therapy (IVOST) and/or complex oral antibiotic therapy is appropriate, and the benefits of early IVOST/complex oral antibiotic therapy along with key considerations that determine implementation of both in OPAT settings. As well as being CPD accredited, the course is recognized by the Royal College of Pathologists. The course is designed to be completed over 3 hours per week for 4 weeks.^20^

## Discussion

This review highlighted the lack of comprehensive, accessible resources aimed at patients, particularly when it comes to general information in the provision of OPAT even in HMIC where OPAT predominates. This is in sharp contrast to peer-reviewed literature and expertise such as how a service should be run, and what the structural, management and clinical pillars of a programme are for HCPs.^5,6,9^

Across the grey literature, the OPAT educational resources identified varied considerably in depth, format and intended audience, with most materials developed within high-income health systems and therefore reflecting the service structures, antimicrobial availability and infusion technologies typical of HIC and HMIC settings. Webinars and conference recordings from established OPAT programmes in high-income countries predominantly showcased mature service models and advanced stewardship practices, offering principles that may be transferable but not directly replicable in LMIC or LI contexts. Patient-facing materials, particularly those demonstrating device-specific administration techniques, were inherently aligned with equipment and medicines more accessible in HIC systems, although concepts of safe handling and home-based care remain broadly applicable. Online courses provided structured, accredited training but were largely found in HIC practice. Collectively, these findings indicate that whereas there are some freely accessible OPAT educational material, its direct applicability varies across income settings, underscoring the need for context-specific, adaptable educational tools to support the global expansion of OPAT services.

The patient educational materials identified are helpful but there are limitations in accessibility, scope, customization, language availability and interactivity that may also limit application, especially to LMICs. It was noted during the review that while some organizations, industry sources and home infusion companies do provide information, it is often very specific and not easily adaptable for other purposes. Most resources identified in this review represented expert opinion rather than formally peer-reviewed or accredited materials, with only the FutureLearn OPAT and IVOST courses meeting criteria for accredited, peer-reviewed educational content. The success of OPAT programmes depends not only on clinical management but also on the ability of patients and healthcare teams to communicate and understand the nuances of the treatment process and various care models. This is especially beneficial for resources-limited areas such as LMICs where limited healthcare access can make managing complications more challenging. The process of receiving intravenous antibiotics outside a hospital setting can be intimidating for patients. Without clear, easily digestible resources, patients may experience increased anxiety or confusion about the treatment process, such as how to administer the antibiotics, recognize side effects or when to seek help in case of problems. While healthcare professionals typically provide some initial guidance and counselling, the lack of ongoing patient education materials can lead to poor follow-up care and complications.

To address the lack of resources and improve the OPAT experience for both patients and healthcare providers, several steps could be taken within the international OPAT community involving patient advocacy groups and established societies. A summary of recommendations is provided in Table 1. Developing clear, evidence-based information resources about OPAT for both patients and healthcare professionals would help ensure consistency in care delivery. These could include guides on common conditions treated, the treatment process, medication used, signs of complications, medication administration instructions and contacts for follow-up support. To cater to diverse patient populations and differing countries, resources should be available in multiple languages and in various formats (e.g. print, online, video) with accessible features, such as large fonts or voiceover options. These resources must also consider that standardization may be challenging due to the nuances among service providers. Developing interdisciplinary educational resources that focus on treatment options, patient education and communication strategies will help healthcare professionals deliver more consistent and patient-centred care.

**Table 1.** Summary of recommendations for OPAT educational resources

Recommendation
Develop guides on common conditions treated in OPAT and the medications used
Develop resources on the treatment process, medication administration, signs of complications and contacts for follow-up support that can be customized
Provide the resources in multiple languages and in various formats (e.g. print, online, video) with accessible features such as large fonts or voiceover options to cater to diverse patient populations
Develop a process to support peer review and sharing across countries and settings

Several limitations should be acknowledged in this scoping review. First, the search was restricted to resources published in English and sourced from a predefined list of grey-literature databases. This approach may have excluded relevant materials published in other languages or hosted on platforms not captured within our search strategy. Given the global variation in OPAT service development, language restrictions may have disproportionately limited representation from low- and middle-income countries. Second, although efforts were made to conduct a comprehensive search of grey literature, the use of web-based search engines introduces challenges. Search engine algorithms are dynamic and user-specific, making searches difficult to reproduce and potentially introducing bias in the resources identified. Differences in browser cookies, geographic location and real-time algorithm changes may all have influenced the search outputs. Third, stakeholder consultation yielded a relatively low response rate from international colleagues. While consultation is recommended within scoping review methodology to enhance relevance and contextual interpretation of findings, limited engagement may have led to underrepresentation of certain regions or OPAT service models. This may have particularly affected insights from countries where OPAT resources are less formalized or where dissemination occurs outside major professional networks. Fourth, because grey literature lacks standardized indexing and consistent quality appraisal mechanisms, there is a possibility that additional relevant resources exist but were not identified despite manual screening of titles and content. Fifth, this review focused on free-to-access resources. Although this aligns with the objective of understanding what is readily available to patients and healthcare professionals, it means that subscription-based or institution-restricted educational materials were not included. As a result, the findings reflect only the open-access landscape and may not fully represent the breadth of OPAT educational tools currently in use internationally. Finally, the impact and reach of the identified educational resources could not be assessed, as data relating to use, learner engagement and effects on clinical practice were not consistently available.

### Conclusions

Overall, while most resources were developed within high-income healthcare systems, many offer adaptable principles that can support OPAT practice development in LMIC settings, particularly where foundational stewardship and outpatient care structures are emerging. However, the lack of apparent resources aimed at general OPAT information represents a missed opportunity in patient care and education. Without comprehensive and accessible materials, patients may struggle to fully understand their treatment, leading to poor adherence and increased complications. Likewise, healthcare professionals may face challenges in providing effective care without educational resources. Addressing these educational gaps is crucial for the success of OPAT programmes and better patient outcomes. We call on societies and patient advocacy groups to work together to address this educational gap. Involving the patient at the heart of OPAT programmes is central to service design and overall patient safety.

## Transparency declarations

The following authors have been involved in the development or delivery of educational resources included in this review: F.S., B.R. (The University of Queensland: OPAT Masterclasses), T.G. (ID Stewardship: An Overview of OPAT Practices), M.G. (FutureLearn OPAT and IVOST courses). M.G. is co-chair of the BSAC OPAT initiative. These resources were assessed using the same predefined methodology and evaluation criteria as all other included resources. Some authors may also have contributed individual presentations to conference archive resources included in the review. Such contributions were not considered equivalent to involvement in the development of the educational resource itself.
